# Relationships between Long-Term Demography and Weather in a Sub-Arctic Population of Common Eider

**DOI:** 10.1371/journal.pone.0067093

**Published:** 2013-06-21

**Authors:** Jón Einar Jónsson, Arnthor Gardarsson, Jennifer A. Gill, Una Krístín Pétursdóttir, Aevar Petersen, Tómas Grétar Gunnarsson

**Affiliations:** 1 University of Iceland, Research Centre at Snæfellsnes, Stykkishólmur, Iceland; 2 Institute of Biology, University of Iceland, Reykjavík, Iceland; 3 School of Biological Sciences, University of East Anglia, Norwich, United Kingdom; 4 Icelandic Institute of Natural History, Garðabær, Iceland; 5 University of Iceland, South Iceland Research Centre, Selfoss and Gunnarsholt Hella, Iceland; Norwegian Polar Institute, Norway

## Abstract

Effects of local weather on individuals and populations are key drivers of wildlife responses to climatic changes. However, studies often do not last long enough to identify weather conditions that influence demographic processes, or to capture rare but extreme weather events at appropriate scales. In Iceland, farmers collect nest down of wild common eider *Somateria mollissima* and many farmers count nests within colonies annually, which reflects annual variation in the number of breeding females. We collated these data for 17 colonies. Synchrony in breeding numbers was generally low between colonies. We evaluated 1) demographic relationships with weather in nesting colonies of common eider across Iceland during 1900–2007; and 2) impacts of episodic weather events (aberrantly cold seasons or years) on subsequent breeding numbers. Except for episodic events, breeding numbers within a colony generally had no relationship to local weather conditions in the preceding year. However, common eider are sexually mature at 2–3 years of age and we found a 3-year time lag between summer weather and breeding numbers for three colonies, indicating a positive effect of higher pressure, drier summers for one colony, and a negative effect of warmer, calmer summers for two colonies. These findings may represent weather effects on duckling production and subsequent recruitment. Weather effects were mostly limited to a few aberrant years causing reductions in breeding numbers, i.e. declines in several colonies followed severe winters (1918) and some years with high NAO (1992, 1995). In terms of life history, adult survival generally is high and stable and probably only markedly affected by inclement weather or aberrantly bad years. Conversely, breeding propensity of adults and duckling production probably do respond more to annual weather variations; i.e. unfavorable winter conditions for adults increase probability of death or skipped breeding, whereas favorable summers can promote boom years for recruitment.

## Introduction

Global climate change is predicted to increase the Earth’s mean annual temperature by 2–4°C in the next decades [1], [2] and influence the frequency of weather extremes, such as severe storms and droughts. The resulting impacts on animal populations are likely to be significant at both regional and global scales [3], [4], [5], [6], [7], [8], [9].

In several migratory bird species, the timing of spring migration and subsequent initiation of breeding have been shown to occur progressively earlier in response to weather changes [6], [7], [10], [11]. Among long-lived seabirds, changes in local weather conditions have also been shown to influence nest initiation dates [12] and the subsequent timing of prey availability for nestlings [13], chick growth and fledging rates [14], [15] and adult survival rates [16]. Extreme weather events can also strongly influence populations [17], [18], [19], although their relative rarity can make their effects difficult to quantify. Among bird populations, prolonged periods of severe weather, successive harsh winters, prolonged droughts or episodic weather events such as storms or hurricanes can directly influence breeding success or mortality rates [20], [21]. Altered frequencies of episodic weather could thus be a key mechanism affecting population-level responses to climatic changes.

Arctic and sub-arctic nesting species are particularly vulnerable to global climate change because climate will change disproportionately more at the highest latitudes [21], [22]. The short summer at northern latitudes predisposes species to complete breeding in a particularly short time and renesting potential is more limited for Arctic-nesting species than it is for species nesting at temperate latitudes [23]. For long-lived species with high adult survival and low reproductive output, the long-term data series which are necessary to reveal the effects of weather conditions on fitness are rarely available.

Common eider *Somateria mollissima* in Iceland nest under sub-arctic weather conditions: and thus, can experience relatively pronounced weather fluctuations [24], [25]. Previous studies have indicated that weather conditions in the months prior to nesting can influence breeding numbers, first nest initiation dates, nest site selection, clutch sizes and nest success [26], [27], [28], [29]. Weather conditions can also influence eider body condition and phenology because: 1) this species uses endogenous reserves for incubation [30], [31]; 2) is a short-distance migrant, both breeding and wintering at high latitudes [26], [27], [32] and so is exposed to high latitude weather conditions year round; and 3) environmental variation (such as weather) can induce physiological changes which can manifest as differing breeding trade-offs, such as nest site selection, nest desertion, yolk hormone levels and immune function [31], [32], [33]. Previous studies have shown that the North-Atlantic Oscillation index [34] (hereafter NAO) is positively correlated with body condition of breeding female common eider and the proportion of juveniles in hunting bags [31], [35]. These correlations with regional-scale NAO indices are likely to result from local-scale effects of weather conditions on duckling productivity and/or recruitment but the details of these relationships are unknown. Lastly, effects of weather may vary within breeding seasons; unfavorable (cold/wet or warm/dry) conditions early in spring may negatively affect nest success whereas favorable conditions in late spring (warm/wet) can positively affect nest success [29].

We collated information on nest numbers for 17 different eider colonies spanning 30–100 years. Our objective was to determine: 1) the spatial scale at which weather conditions may be associated with nest numbers of eiders within and between colonies, i.e. determine if relationships with weather occur consistently in all colonies; 2) whether local weather conditions influence eider nest numbers directly (effects within same year, i.e. lag = 0) or via recruitment (lagged effects 2–3 years later); and 3) the effects of rare episodic weather events on nest numbers in subsequent years. Improved understanding of the potential significance of episodic or extreme weather events to bird numbers is particularly important in light of present climate change predictions. These long-term data span generations of the long-lived common eider and encompass a wide range of weather conditions. Thus, these data provide an unusual opportunity to explore the short and long-term effects of both rare extreme weather events and more subtle weather variation on population processes at different spatial scales.

Climatic models for the Arctic and sub-Arctic regions generally not only predict increased mean temperatures and precipitation, but also more variation in these parameters [36]. Regarding predictions for effects of weather on eiders, we paid special attention to two possible scenarios, both of which are predicted for Iceland in future climate predictions for the next 50–100 years [37]. Firstly, we considered increased winter mildness, which may offer a mixed blessing for common eiders [38]: while common eider females arrive in better body condition at breeding grounds following warmer winters [35], their favorite winter food, blue mussels (*Mytilus edulis*) provide a better food supply during colder winters [39]. Secondly, we considered the effect of summer wetness on recruitment, because wet summers are negative for duckling production via increased predation [40]. Therefore we predicted 1) that nest numbers increased following milder winters because of the generally better body condition of females; or 2) that nest numbers decreased following milder winters because of reduced food supply; 3) that a negative effect on recruitment was observed in the abundance of nesting females 2–3 years after wet summers; 4) that aberrantly cold years or harsh winters had a greater effect on common eider nesting abundance than more normal variation in weather conditions.

## Methods

### Ethics Statement

We used existing data, which were sent to us from eider farmers at 17 locations in southwest, west, northwest and north Iceland. Common eider are a wild and free-ranging species in Iceland, the down is collected from the nests during late incubation or after the birds have left the colonies [41]. Down collection during late incubation does not affect incubation temperatures [42]. Eider farming is regulated by Icelandic law no. 84/1989, for the purpose of which common eider is protected by law (currently no. 64/1994) from hunting and egg collection. The common eider has been completely protected from hunting in Iceland since 1849 and from egg collection since 1787 because of the economic importance of down collection [41]. However, the effects of the protection act on population dynamics, or any other population changes during the 18th or 19th century have not been quantified for this population.

### Study Area

#### Climate

Iceland has a relatively mild climate relative to its latitude because of the tempering effect of Atlantic Ocean currents, which flow along the southern and the western coast [25]. However, the climate is sensitive to changes in storm occurrence with mild Atlantic air coming into contact with cooler Arctic air. Rainfall generally is higher in the southern and western parts than in the northern part of Iceland. A positive North Atlantic Oscillation Index (NAO), which indicates a prevailing western wind in the Northern Atlantic, often means increased precipitation whereas NAO has no relationship with temperature in Iceland [43].

The average temperature in Iceland increased by 0.7°C per century since the early 1800s [37]. The warming during the 20^th^ Century was comparable to the global climate change, although since 1975, annual mean temperature has increased by 0.35°C, which is somewhat greater than the global average increase. It is predicted that the annual mean temperature will increase by 1°C (0 to 2°C) by 2050. Precipitation in Iceland likely will increase during the 21st century by 0.4–0.8% per decade or 2–3% per every 1°C of increased temperatures [37]. Predicted future warming will be most pronounced during the winter but least during the summer.

#### Study species

The common eider is Iceland’s largest waterfowl population and not listed on the Red List (bird species listed as LR by IUCN standards, or of greater concern) issued by the Icelandic Institute of Natural History [44]. The estimated breeding population size is 300.000 pairs, with a wintering population of 847,000 (566,000–1,127,000, 95% confidence limits) individuals [44], [45]. Nest initiation begins 1 May at the earliest but the last nests are initiated in mid-June. Clutch size generally is 3–5 eggs which is similar to that in other populations [26], [41], [42], [46], [47], [48]. Earlier nesting is related to increased clutch sizes [26], [35], [49]. In Iceland, male eiders remain with females by their nests into mid-incubation, unlike that reported for other populations [50]. Many common eider colonies have been husbanded for eider down collection since before the 19th century, often maintained by the same families who have kept records of numbers of nests throughout the 20th century.

### Data Collection

We collated data on the number of nests per year (hereafter breeding numbers) from 17 common eider colonies from southwest, northwest and north Iceland (Fig. S1). The length of the time series differed between colonies; the longest spans 101 years (1906–2007) and the shortest spans 26 years (1981–2007) (Table 1). Nests were counted in a standardized way by experienced observers (eider farmers) who survey in great detail in order to ensure that every nest is located, as the eider down that they gather from the nests is a source of important revenue.

**Table 1:** Common eider colonies, location, series length (years), relevant weather stations and covariance structures used to estimate correlations between breeding numbers and local weather conditions, for common eider *Somateria mollissima* breeding in Iceland.

	Colony	Area	Series length	Series length used in analysis	No. years	Weather	Weather station(s)	Covariance structure
1	Rifgirðingar	Breiðafjörður	1901–1930	1901–1930	29	t, p, r	Stykkishólmur	none
2	Brokey Islands	Breiðafjörður	1906–2007	1906–2007	101	t, p, r	Stykkishólmur	none
3	Lækur	West Fjords	1953–2007	1961–2007	46	t, p, r, f	Stykkishólmur, Bolungarvík	none
4	Bjarneyjar	Breiðafjörður	1958–2007	1961–2007	46	t, p, r, f	Stykkishólmur	AR 1,2
5	Svefneyjar	Breiðafjörður	1958–2007	1961–2007	46	t, p, r, f	Stykkishólmur	MA1,1
6	Hrísey	North Iceland	1960–2007	1961–2007	46	t, p, r, f	Stykkishólmur, Akureyri	none
7	FV-NK	SW Iceland	1961–2007	1961–2007	46	t, p, r, f	Stykkishólmur, Reykjavík	none
8	Laxamýri	North Iceland	1968–2007	1977–2007	30	t, p, r, f	Stykkishólmur, Akureyri	none
9	Inneyjar	Breiðafjörður	1974–2007	1977–2007	30	t, p, r, f	Stykkishólmur	none
10	Úteyjar	Breiðafjörður	1974–2007	1977–2007	30	t, p, r, f	Stykkishólmur	none
11	Rif	Breiðafjörður	1975–2007	1977–2007	30	t, p, r, f	Stykkishólmur	none
12	Tjörnin	SW Iceland	1977–2007	1977–2007	30	t, p, r, f	Stykkishólmur, Reykjavík	none
13	Skáleyjar	Breiðafjörður	1977–2007	1977–2007	30	t, p, r, f	Stykkishólmur	MA1,1
14	Flatey	Breiðafjörður	1977–2007	1977–2007	30	t, p, r, f	Stykkishólmur	AR1,1
15	Þyrill	SW Iceland	1978–2007	1978–2007	29	t, p, r, f	Stykkishólmur, Reykjavík	none
16	Bíldsey	Breiðafjörður	1978–2007	1978–2007	29	t, p, r, f	Stykkishólmur	none
17	Sauðeyjar	Breiðafjörður	1981–2007	1981–2007	26	t, p, r, f	Stykkishólmur	none

t = temperature (°C), p = atmospheric pressure (ppt), r = precipitation (mm), f = wind speed (m/s).

**Figure 1:**
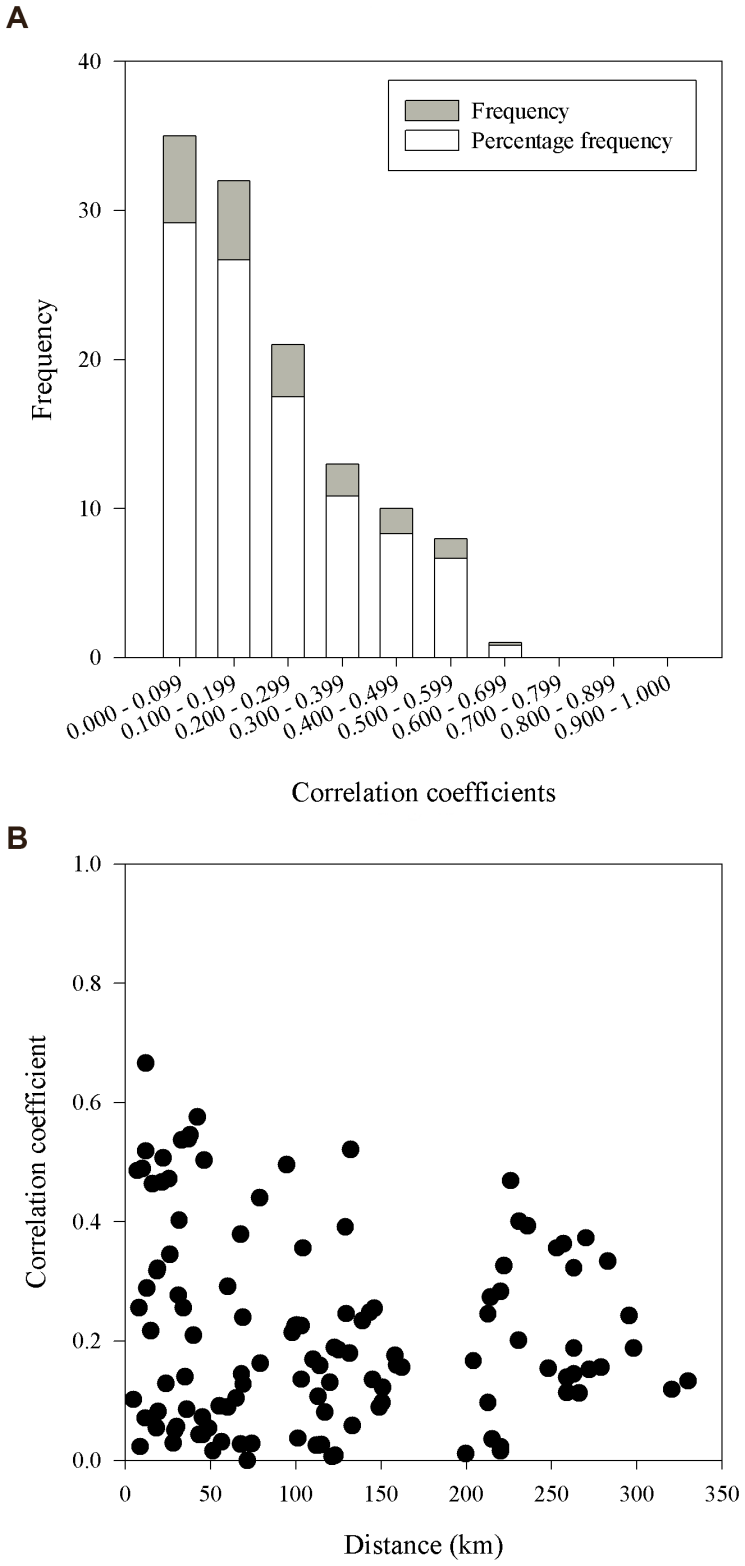
Frequency distribution of common eider population synchrony. Synchrony was measured by pair-wise cross-correlation coefficients of breeding numbers with lag = 0, among 16 colonies (120 correlations) in Iceland 1977–2007. Low synchrony was inferred because of 120 pair-wise correlations, 88 (73%) had a cross-correlation coefficient <0.3, which were not significant at the α = 0.05 level. The mean correlation coefficient was 0.213; with the highest correlation coefficient = 0.666.

Weather parameter f was unavailable until 1949. Numbers in far left column refer to locations in Figure 1. Colony FV-NK is Fuglavík-Norðurkot.

All data come from colonies which are used for eider down collection. The high revenues available from eider down mean that farmers typically undertake management aimed at encouraging high nesting densities, such as controlling numbers of avian and mammalian predators, and providing nest shelters. The extent of management activities differs between colonies, regions, owners or care-takers, and generations of owners, and depends on local conditions. However, there is no indication that variation in colony management varies systematically with weather conditions.

### Statistical Procedures

Our analyses involved five types of statistical procedures. We primarily used time-series analyses, including 1) autoregressive integrated moving averages analysis; 2) pair-wise cross-correlation analysis; and 3) impact analysis with ARIMA noise models and deterministic intervention functions. The first two were used to determine if there were correlations between time-series of weather and nest counts. Impact analysis was used to determine if individual years caused marked changes in the nest counts, such as turns, interventions or new trends. Prior to running the time-series analysis, we used 4) principle components analysis (PCA) to reduce the number of weather variables and create linear indices (PC scores) of types of seasons (for example, “rainy summers” or “cold, windy winters”). Lastly, we used 5) false discovery rates significance thresholds (which lowers P-value thresholds below α = 0.05, scaled with number of comparisons made) to consider all correlations in unison to avoid reporting spurious results.

Our analysis of population synchrony and correlations between weather and breeding numbers followed a time-series approach [51], using autoregressive integrated moving average analysis developed by Box and Jenkins (PROC ARIMA) [51], [52]. This method requires that series are de-trended or differenced so that each series has a constant dispersion around a single mean (hereafter stationarity). We first tested series for non-stationarity using the Dickey-Fuller test and found that all series only became stationary following differentiation [51]. In most cases, the models fit a given series without a covariance structure. Where needed, as indicated by examination of autocorrelation functions, the covariance structures used were either, first- and second order autoregressive (AR1,1 & AR1,2 ) or first-order moving averages (MA1,1) (Table 1).

#### Population synchrony

We assessed the possibility of population synchrony in our data since we used 17 colonies between 4 and 330 km apart, and a high level of synchrony could indicate the presence of a population driver operating on a larger scale, such as weather conditions. Following Ranta et al. [53] and Koenig [54] we used a comparison of the temporal dynamics of common eider nests in the 16 colonies spanning 1977–2007, i.e. the 31 years in which all colonies had available data. We then made all pair- wise comparisons, using cross-correlation analysis with time lag = 0 among the 16 colonies, yielding 120 cross-correlation coefficients. We used PROC ARIMA in SAS to obtain cross-correlation coefficients between each pair of colonies. To estimate the degree of synchrony, we: 1) compared our distribution of correlation coefficients (R) to those of Ranta et al [53]; and 2) plotted a Mantel graph [54], where distance between all pairs of colonies are plotted against the respective correlation coefficients. We interpreted results as follows: correlation coefficients 0.5 or higher were interpreted as indicating a high degree of synchrony between the respective colonies, whereas values less than 0.3 (P = 0.05 at approximately R = 0.35) were interpreted as indicating relatively little synchrony between colonies. Note that a significant relationship between distance (km) and correlation coefficients also would suggest synchrony in the data, but that a lack of such relationship does not necessarily indicate an absence of synchrony.

#### Relating weather to breeding numbers

We examined the effects of variation in local weather conditions on breeding numbers by analyzing cross-correlations with indices of local weather [26], [28], and the regional-scale mid-winter NAO-index, in PROC ARIMA. In order to explore the effects of weather conditions during each of the four seasons, monthly averages of local weather data (temperature, precipitation, atmospheric pressure and wind speed) were averaged for each season (winter: December-March; spring: April-May; summer: June-September; autumn: October-November) and then indexed using a principal components analysis (PCA). The PCA not only reduced number of variables within each season but, more importantly, accounted for combined effects and interplay of weather variables within each season [26], [28], [55]. PC scores that explained ≥25% of the variation in the four weather variables were included in subsequent correlations with eider demography.

We used the weather station in Stykkishólmur, West Iceland, which is representative for all of Iceland [24], [56] and most of our data (11 of 17 colonies) come from nearby colonies in the bay of Breiðafjörður, West Iceland (Fig. S1). However, we also examined relationships using weather data in the vicinity of six colonies furthest from Stykkishólmur (see Table 1). Weather recording in Stykkishólmur began in 1846 [25] but data on wind speed were not available until spring of 1949. Thus, our correlation analysis of local weather parameters included only temperature, precipitation and atmospheric pressure for these periods (Table 1). For the 101 year long Brokey Islands series, we compared analyses of: 1) the period 1906–2007 with three weather variables; and 2) two analyses: 1901–1950 with three weather variables and 1951–2007 with four weather variables (Table 1). These approaches yielded very similar results and thus the first results from 1906–2007 are presented for Brokey Islands.

Time series starting at similar times were analyzed from a common starting date (1961; 5 series, 1977; 10 series, Table 1) for consistency. Upon detecting a significant cross-correlation at the 5% level for at least one of 17 colonies, we used the corresponding P-values from all the colonies to calculate the False Discovery Rate significance thresholds (FDR) [57] against which P-values of tests were evaluated. Here, we present findings from the classical one-stage method for FDR, following [57]. Finally, we used P-values 1) from both available colonies when calculating FDRs for 1900–1930, 2) from all six available colonies when calculating FDRs for 1961–2007 and 3) from all 16 available time-series when calculating FDR for the period 1977–2007.

#### Time-lagged relationships between weather and breeding numbers

Common eiders start breeding at 2–3 years of age [58] and female common eiders are highly philopatric to the maternal breeding area [59], [60]. Consequently, changes in the size of breeding colonies, as a consequence of weather effects on duckling production, may not be apparent for up to three years. Based on the recruitment age of the common eiders, we considered 0 (no lag), 1, 2, and 3 year lagged effects of local weather on breeding numbers, to explore effects of weather on recruitment [27]. We saw no biological reason for weather to be correlated with eider numbers at lag 4–5 years or later.

#### Impact analysis of severe weather events

Episodic weather events (i.e. severe or unusual weather that persists for a limited length of time) can potentially affect breeding numbers for years after a weather event. Prolonged periods of severe weather (i.e. harsh winters or droughts) or episodic weather events may cause widespread breeding failure or mortality and can result in population declines [20]. However, such single, possibly rare, events may not be indicated by a correlation analysis on a long time-series. Therefore, we used impact analysis on breeding numbers, following [51] who considered effects of single events (interventions, or “shocks to the system” [61]). For example, the winter of 1918 was the most catastrophic weather event during the 20th Century in Iceland. Impact analysis assesses the response in a time-series to discrete events or interventions that occur perhaps once during the span of a time-series. Impact response models are formulated as a regression function, with a response series as a dependent variable, and the regression model independent variables comprised by an ARIMA noise model and a deterministic (dummy variable for the impact event) intervention function [51].

Here, years of potential impact are coded as 1, whereas all other years are coded as zeros. We selected years for impact analysis based on NAO and historical years. For the NAO, we considered using the standard deviation (SD), multiplied by 2, which is a common practice to select “extreme” values. The 2×SD was 3.87 for 1990–2007 (the combined study period). However, when we compared NAO years with other historical weather events, we noted that NAO years of ≈3.3 to ≈3.7 coincided with these other weather events and thus, generally were remarkable enough to be considered. Thus, we settled on NAO values of 3.3 or greater to be inserted as impact years (1903, 1983, 1989–1990, and 1992) as well as negative values of −3.3 or lower (1917, 1936, 1963, 1969, and 1996). We then added remaining historical weather events [25]; the very cold year of 1918, the years 1965–1971 & 1979 during which sea-ice was unusually close to Iceland, and the warmer years of 1928–1946 [56]. Successive impact years (for example 1917–1918 and 1994–1995) were treated as a single impact event.

#### Effects of density-dependence

Lastly, we tested for possible density-dependence in breeding numbers by examining correlations between breeding numbers in year y with those in years y+1 to y+4. According to this approach, a significant cross-correlation would indicate that density-dependence was operating within each of the colonies. As before, we used PROC ARIMA to test for these relationships.

## Results

Inter-annual synchrony between colonies was low. Of 120 pair-wise correlations, 88 (73%) had a cross-correlation coefficients <0.3 (Fig. 1a), indicating low synchrony between colonies. Of the remaining 32 cross-correlation coefficients, 26 were significant at the α = 0.05 level (cross-correlation coefficients ≥0.35), indicating high synchrony between colonies. The mean correlation coefficient was 0.213; with the highest correlation coefficient = 0.666. The Mantel graph (Fig. 1b) showed a highly variable distribution of correlations with respect to distance. In itself, the lack of a correlation did not mean that synchrony is low. Due to the overall low synchrony between Icelandic eider colonies, we did not pool any of the 17 time-series.

**Figure 2:**
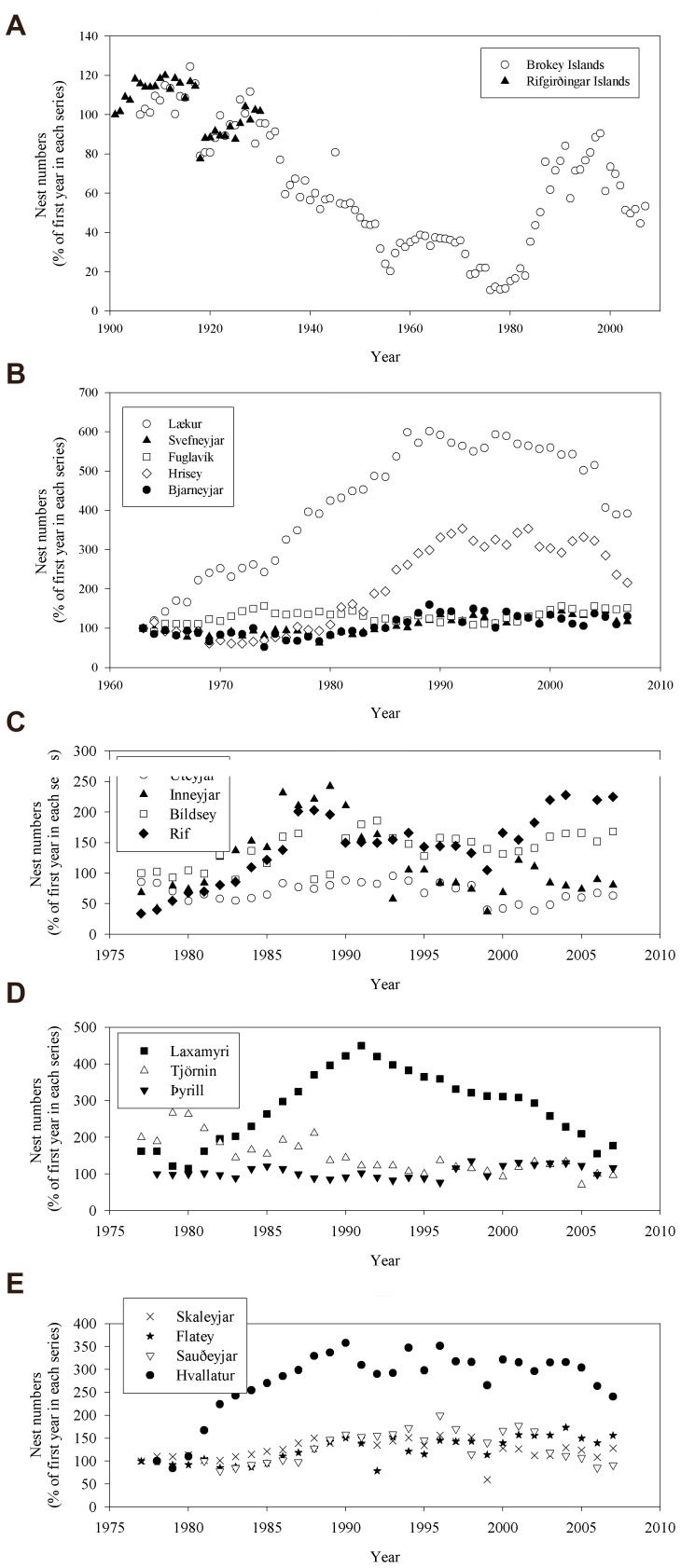
Variation in the numbers of breeding common eider *Somateria mollissima* in 17 colonies in Iceland. Breeding numbers are shown during (A) 1901–2007, (B) 1963–2007, (C, D, E) 1978–2007. Y-axis shows breeding numbers which were standardized by setting the first year of each series at 100%. Note the differing scales on y- and x axis.

The trends in numbers of breeding common eiders across the 17 colonies were variable, but several colonies showed a trend for increasing numbers after the 1960s or 1970s and also experienced declines in numbers from the 1990s onwards (Fig. 2). The two oldest time series, Rifgirðingar and Brokey Islands began in 1901 and 1906 respectively, and both remained stable at around 500–1000 pairs until 1918 when both populations crashed to ∼60% of their previous peak number before steadily increasing again until 1930 (Fig. 2).

### Cross-correlations of Breeding Numbers with Local Weather

**Figure 3:**
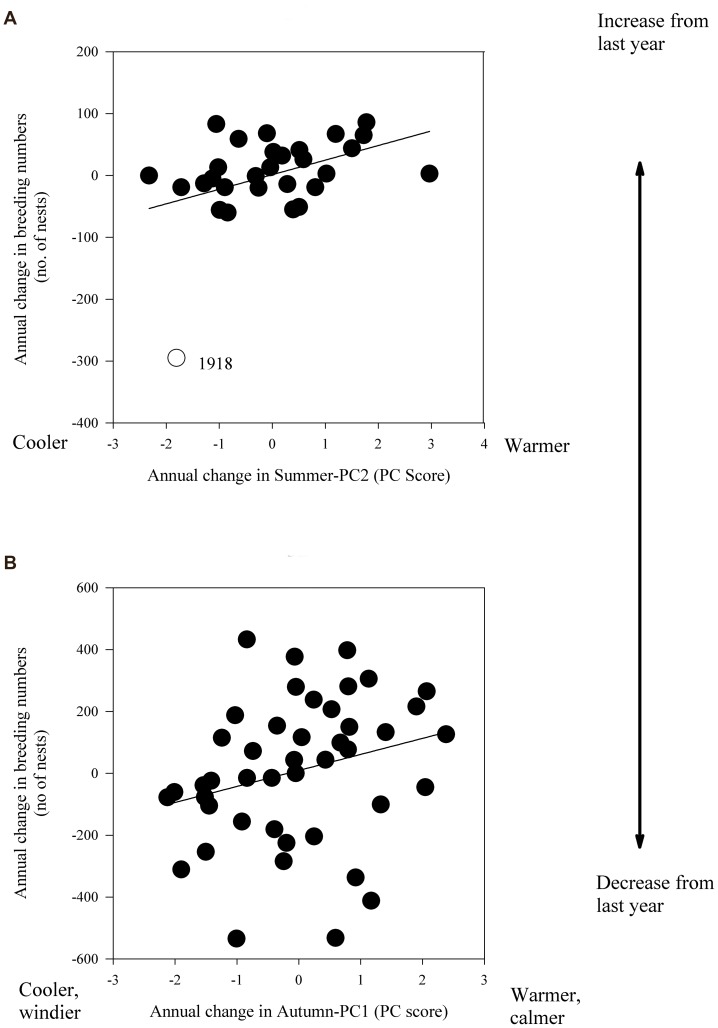
Relationship between annual changes in breeding numbers and weather. In A) summer weather (see PC score loadings in Table S1) and breeding numbers (no. of nests) of common eider at Rifgirðingar, West Iceland 1900–1930; B) autumn weather and breeding numbers of common eider at Svefneyjar, West Iceland 1961–2007. Note the differing scales on y- and x axis.

We found only two cross-correlations between local weather and breeding numbers within 1 year (lag = 0). Firstly, at Rifgirðingar, there was a significant correlation between summer weather conditions and breeding numbers (Summer-PC2, t = 2.30, P = 0.02, FDR-derived significance threshold = 0.025), with warm previous summers (positive PC scores, Table S1) being associated with greater breeding numbers (Fig. 3A) and cooler previous summers associated with decreased breeding numbers. Between 1919 and 1930, the population at Rifgirðingar had not recovered from the sharp decline that occurred in 1918 (see also impact analysis below). Secondly, in Svefneyjar (but not in any of the other colonies, including neighbouring colonies) final models for breeding numbers included a positive correlation with autumn weather (Autumn-PC1, t = 2.78, P = 0.0054, FDR-derived significance threshold = 0.0071). Autumn-PC1 indicates that a relatively warm and calm autumn prior to breeding (loadings 0.74 on temperature and −0.61 on wind speed, Appendix 2) was positively correlated with breeding numbers (Fig. 3B), whereas lowered breeding numbers followed cooler, windier autumns.

In Brokey Islands, the longest time-series, there were no relationships between variation in local weather conditions and breeding numbers. We included this series within all time periods considered in this study because of its length. Correlations with weather for Brokey Islands produced the same non-significant results, regardless of the periods considered or whether the PC scores were based on three weather variables (1906–2007 full series, 1906–1930 for comparison with Rifgirðingar, 1906–1949) or four weather variables (1950–2007).

**Figure 4:**
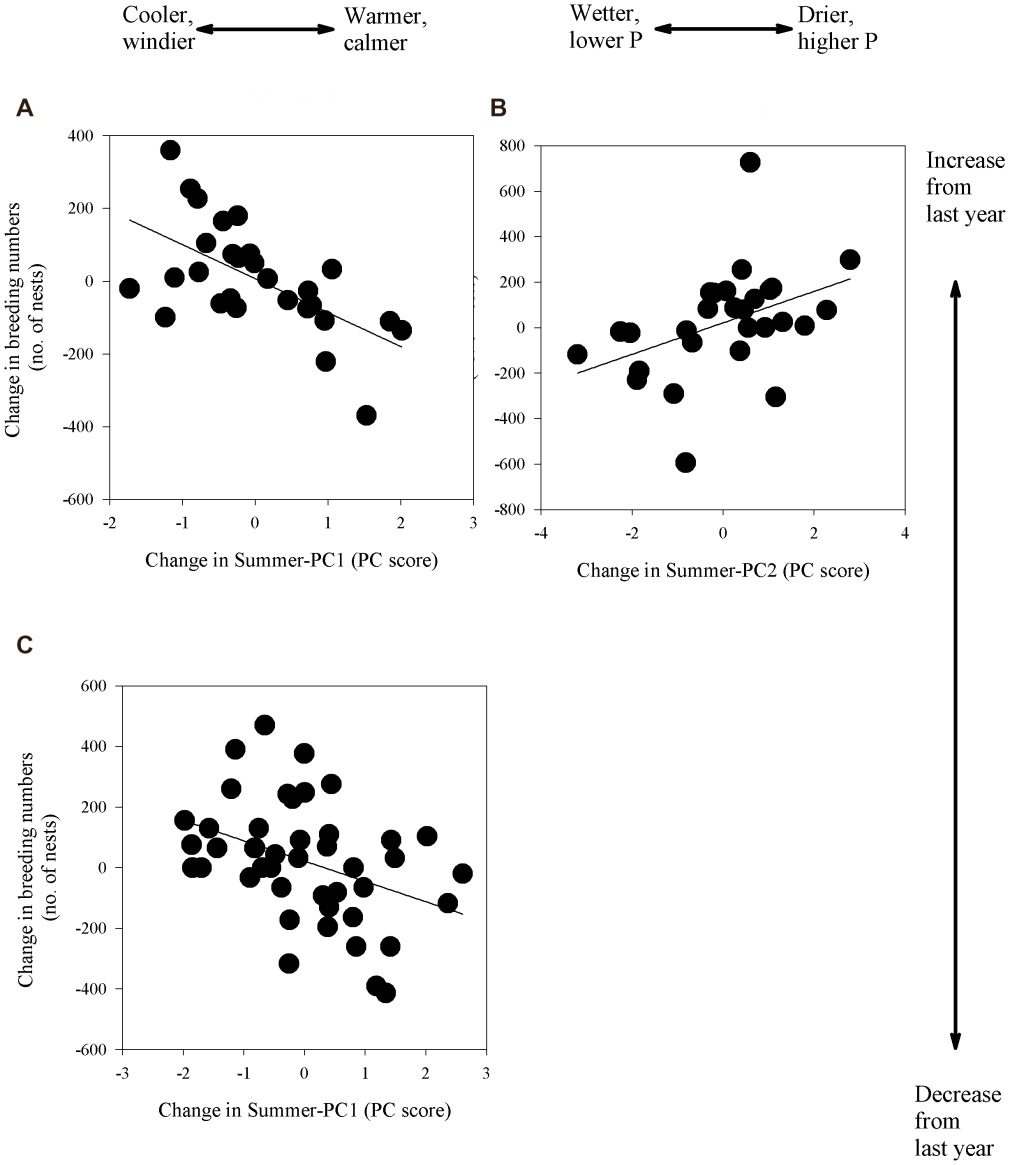
Relationship between summer weather and breeding numbers (no.of nests) 3 years later. Shown are three common eider colonies in Iceland 1977–2007 (A, B) and 1961–2007 (C). Graphs on the left-hand side have Summer-PC2 on the X-axis whereas graphs on the right-hand side have Summer PC1 on the X-axis (see explanations on top of each column of graphs). Note the differing scales on y- and x axis.

We found few lagged (breeding numbers 1–3 years after weather) correlations between local weather (indexed by the PCA) and breeding numbers within colony. However, in three colonies, there were significant correlations between weather in a given summer and breeding numbers recorded 3 years following that summer (Fig. 4). There were two kinds of lagged correlations with summer weather: 1) a positive effect of Summer-PC2 (where positive PC scores indicated higher pressure, drier summers 3 years previously and negative scores indicated lower pressure, wetter summers; see Table S1) on breeding numbers three years later at Flatey 1977–2007 in Breiðafjörður (Fig. 4B t = 3.84, P = 0.0001, FDR-derived significance threshold = 0.0031) and 2) a negative effect of Summer-PC1 (where positive PC scores indicated warmer, calmer summers 3 years previously and negative scores indicated cooler, windier summers; see Table S1) on breeding numbers three years later at Þyrill 1977–2007 (t = −3.87, P = 0.0001, FDR-derived significance threshold = 0.0031) in southwest Iceland (Fig. 4A) and at Hrísey 1961–2007 in North Iceland (Fig. 4C); Hrísey (t = −2.79, P = 0.0053, FDR-derived significance threshold = 0.0063).

**Figure 5:**
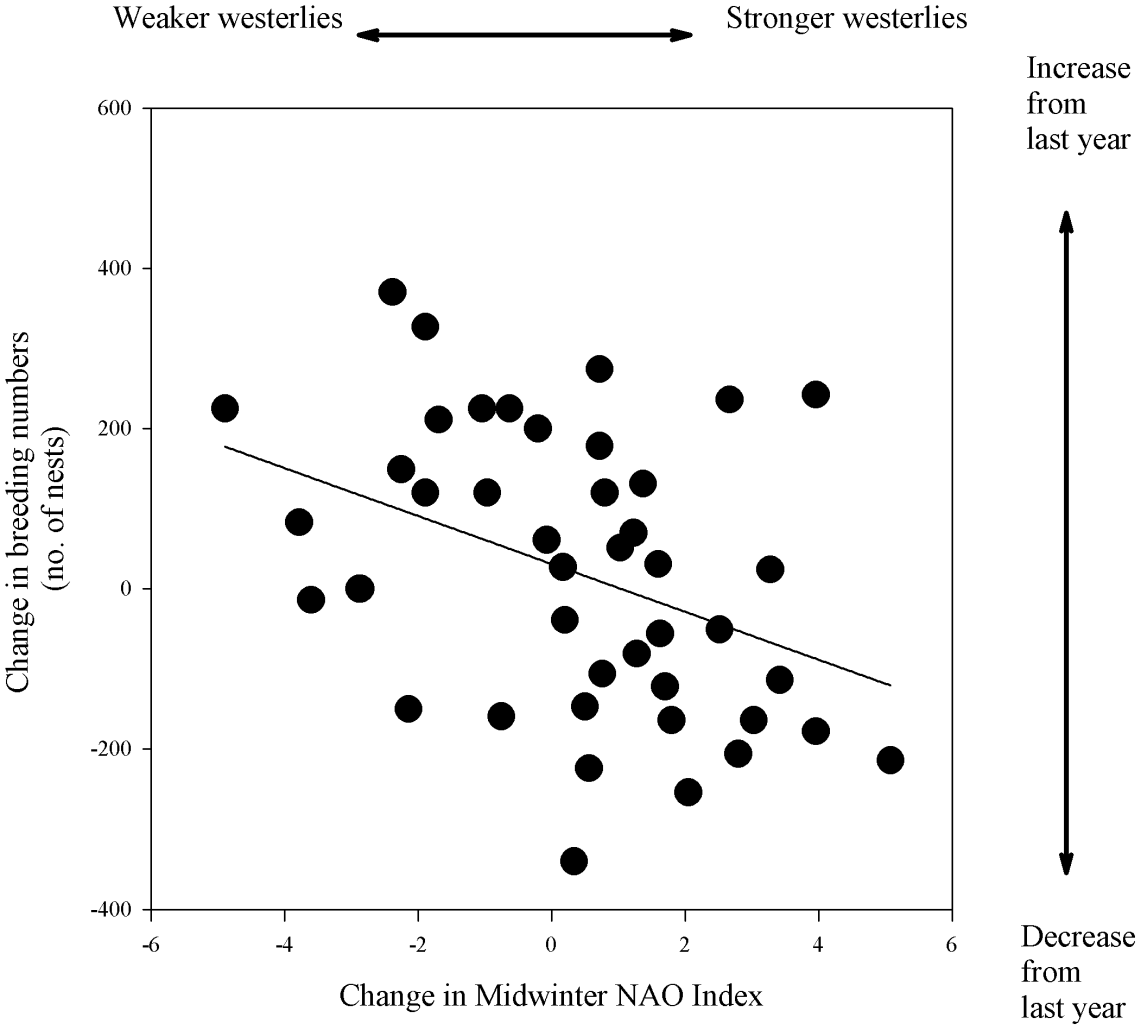
Only one colony showed a relationship with NAO. Relationship between mid-winter (DJFM) North Atlantic Oscillation index and breeding numbers (no. of nests) of common eider at Fuglavík-Norðurkot in southwest Iceland 1961–2007.

We found no correlations between midwinter NAO and breeding numbers for 1901–1930 (Rifgirðingar and Brokey Islands), 1906–2007 (Brokey Islands) or for series ranging 1961–2007 at lags 0–3 years, except for Fuglavík-Norðurkot where a 3-year positive lagged effect was found (t = 2.72, P = 0.0066, FDR-derived significance threshold = 0.0071) (Fig. 5). For the set of series ranging 1977–2007, we found no correlations at any time lags between NAO and breeding numbers for seven colonies.

### Impact of Episodic Weather Events

Years considered as impact years were all numerical NAO values of 3.3 or greater (positive 1903, 1983, 1990, 1992, and 1995; negative 1917, 1936, 1963, 1969, and 1996) and remaining historical weather events in Iceland, including the very coldest year 1918 (see Fig. 3A), sea-ice years of 1965–1971 & 1979 and the warm years 1928–1946.

The impact analysis indicated a series of influential years with aberrant weather affecting breeding numbers in subsequent years (summary in Table 2). The very cold year of 1918 had a strong negative impact in both series that included the year (Rifgirðingar 1900–1930: t = −7.83, P = 0.0001, FDR-derived significance threshold = 0.025; and Brokey Islands 1906–2007: t = −4.48, P = 0.0001, FDR-derived significance threshold = 0.05).

**Table 2:** Summary of impact analyses for 17 common eider colonies in Iceland 1900–2007, which evaluated impact of certain years on breeding numbers of common eider within each colony.

Year entered	No. of series	Weather event	No. of colonies	Colonies impacted
1903	2	High NAO = 3.89	0	
1907	2	Second coldest winter of study period	0	
1917–18	2	Low NAO = −3.80 (1917), coldest year & coldest winter (1918)	2	Brokey Islands, Rifgirðingar
1936	1	Low NAO −3.89	0	
1941	1	The warmest of “warm years” 1928–1946	0	
1963–64	7	Low NAO = −3.60 (1963), warmest winter (1964)	0	
1969	7	The coldest year of the sea-ice years 1965–71, lowest NAO = −4.89	0	
1979	16	The coldest spring & 2nd coldest year of the 20th century	0	
1983	16	High NAO = 3.42; 2nd coldest summer	0	
1990	16	High NAO = 5.08	0	
1992	16	High NAO = 3.30	2	Flatey, Inneyjar
1995–96	16	High NAO = 3.96 (1995), Low NAO = −3.78 (1996)	1	Þyrill
2003	16	The warmest year of the study period	0	

* = numbers in parentheses indicate significant relationships (α = 0.05) which were not significant according to FDR.

In series ranging 1977–2007, two years with extreme NAO values (1992, and 1996; NAO >3.3 or NAO<−3.3) were each indicated at least once but never in any of the longer series (see summary of impact year findings in Table 2). The year 1992 (Yearly NAO higher than 3.3) impacted 1) Flatey 1977–2007: t = 3.85, P = 0.0001 FDR-derived significance threshold = 0.0031; 2) Inneyjar 1977–2007: t = −3.54, P = 0.0004, FDR-derived significance threshold = 0.0063. The year 1996 (NAO lower than −3.3) impacted Þyrill 1977–2007: t = 3.82, P = 0.0001, FDR-derived significance threshold = 0.0031.

### Test for Effects of Density-dependence

There were no relationships found between breeding numbers for year y and y+1, to y+4 within any of the colonies (FDR-derived significance thresholds P = 0.0063 for series 1958–2007 and longer, and P = 0.0031 for 1977–2007). Thus, given the method and data, we report that density-dependence generally was not present in breeding numbers in this study.

## Discussion

Of our four predictions, one was met with a twist, i.e. in that there was a negative effect on nest numbers was observed 2–3 years following warmer, calmer (but not wet, as predicted) summers, albeit only for 2 of 17 colonies. Furthermore, one colony showed a positive relationship with drier summers 3 years earlier. Negative effects of impact years, aberrantly cold years or harsher winters were observed for the cold year 1918 and NAO years in the 1990s. In addition to these relationships, weather effects on common eider in Iceland are important with respect to arrival dates to the breeding colony and the subsequent clutch size [26]. The 3-year time lag suggests that duckling production may affect some local populations via recruitment, although the evidence for such a relationship was limited to 3 of 17 colonies. Weather conditions have been implicated as a synchronizing agent in spatially separated populations [62], but we found limited effects of weather on local population size among relatively unsynchronized sub-populations that breed within 330 km of each other. In this study, the lack of a spatial relationship between distance and correlation coefficients confirms the relatively minor effects of weather on eider nest numbers. We found no relationships between NAO and common eider in Iceland for 16 of 17 colonies, the lone exception being Fuglavík-Norðurkot 1977–2006, see also [27]. According to the impact analysis, the cold winter of 1918 had a significant, catastrophic impact on the eider population whereas the population seemed robust to most other episodic years considered.

Our findings indicate a low level inter-annual population synchrony for common eider in Iceland (lag = 0) during 1977–2007. In comparison, see: 1) Ranta et al. 1997 [53], where 50 of 55 (90.9%) of cross-correlation coefficients were 0.5 or higher and the mean cross-correlation coefficient was 0.716: these findings were interpreted as a high overall population synchrony; 2) Koenig 1999 [54] where most coefficients at shorter distances (left-hand side of figure) ranged 0.50 to 0.70, whereas our ranged 0.0 to 0.50. Separate treatment of colonies in our analysis also is likely to be appropriate because common eiders are highly philopatric with up to 98% of females returning to their natal colony every year [59], [60], [63], [64].

We found no relationships between breeding numbers in year t and later years. Given the method and data, this suggests that density-dependence is generally not evident in nest numbers of the Icelandic common eider population, although further studies, using other variables than nest counts are needed to confirm such a statement. Density dependence is highly relevant to interpretation of recruitment indices, particularly when a time-lag is used as a proxy for recruitment, such as in [27] and this study. If a population is under high density-dependence (for example, there are relatively high nest densities at the colony), low recruitment years matter little because recruits are competing for few open nest sites anyway. Conversely, if density matters little, local breeding numbers can be highly influenced by number of recruits.

### A Locally Important, Lagged Effect of Weather on Recruitment

Effects on bird populations can be classified as 1) tub effects, which influence birds in the non-breeding season; and 2) tap effects, which influence breeding output [65]. To date, reported tub effects on common eider in Iceland operated mostly on arrival date and clutch size via body condition [26]. Here, we report possible, localized tap effects: a 3-year lagged correlation between weather and breeding numbers. We attribute these correlations to variation in duckling production or subsequent recruitment of 3 year old females into the respective breeding populations. Such a relationship should not be observed for any colony unless the number of breeding females is somewhat dependent on recruitment.

Duckling mortality is probably relatively more important than hatching failure in limiting breeding success prior to fledging in common eider [66]. Furthermore, females remain faithful to their natal colony and males pair with females from their natal colony more often than would be expected at random [59], [60]. Thus, colony-specific production will partly determine recruitment to a given colony because both sexes display philopatry, although males do so to a lesser extent or not at all [60], [66].

In Flatey, drier summers were positively related to breeding numbers three years later. At Þyrill and Hrísey, warmer, calmer summers were negatively related to breeding numbers three years later. In Scotland, predation on common eider ducklings was more probable in rainy, windy weather than in fine, calm weather and such weather accounted for over 90% of all variation in duckling survival during eight of nine years [40]. In the Netherlands, duckling production was positively related to high temperatures and “calm” weather at the end of May/beginning of June, although there was considerable scatter in the data [66]. Although weather may affect duckling production, food supply is probably the most important limiting factor [67], [68] and conditions during the breeding season can also influence subsequent return rates of adult breeders [68], [69].

### Life History of Common Eider

Like other sea-ducks, the life-history strategy of common eider is essentially that of a long-lived species, with stable, high adult survival but low annual breeding output, although with a potential for high production during favorable years [29], [46], [70], [71,]. Adult survival generally is 0.87 or higher and constant over time for many populations [28], [72]) although not all of them [73]. Adult survival may have less impact on actual population dynamics than recruitment or duckling survival, which may vary considerably over time [50].

Costs of reproduction can become higher in unfavorable years and individual quality probably varies with environmental conditions [74]. Arctic environments provide common eider with a variable environment in most parts of the species range. However, such environmental stochasticity probably is relatively lower for Iceland, which is relatively sub-Arctic compared to Arctic Canada, Svalbard or Greenland. For example, initiation of nesting in Iceland is independent of sea ice, unlike that in Canada or Svalbard [49] and occurs in early or mid-May, rather than late May or June. Furthermore, duckling production generally seems high in Iceland; in Breiðafjörður 2007–2012 it was 0.4 ducklings/females or higher for all years except 2011 and was 0.6 or higher for three of six years (JEJ unpublished data).

During heightened exposures to avian cholera, large clutches were associated with lower survival of breeding females in East Bay, Nunavut [71]. While avian cholera is thus far unknown to Iceland, a similar but more general pattern of consequences of unfavorable conditions on breeding output may apply to this system. Common eiders are well known to skip breeding in some years, which can be seen as a strategy to preserve residual reproductive value, or to employ a trade-off of costs of current reproduction relative to reduced future opportunities or even reduced survival [46], [74]. In our data, non-breeding years are detectable within certain colonies, and many of those occur simultaneously among colonies. However, long-term implications of such years for population dynamics probably have been limited in Iceland, because during skip years, breeding numbers will decline sharply for one year, and generally return to the previous year’s number one year later, with the time-series usually continuing their previous trend for years to come. The source of environmental stochasticity that prompts such years remains unknown, although reduced food availability, unfavorable climate conditions or disease have been implicated as potential drivers [46] along with weather conditions.

### Why is Weather Important in some Colonies – and not Others?

We observed low levels of inter-annual synchrony in the Icelandic common eider population, i.e. the likely absence of a Moran effect and other synchronizing agents, such as dispersal or a synchronized food supply [62]. Such low levels of synchrony indicate that local effects, perhaps via interactions of weather and local topography or resources, are more dominant in this population. The long life expectancy of this species could however mask changes because of high philopatry and the reproductive tactics among breeding females [46]. The lack of relationships between breeding numbers and weather variables indicates that eider colonies are robust in terms of inter-annual changes, perhaps because females can skip breeding in unfavorable years to return in later years.

Despite having limited effects on breeding numbers, weather can affect the arrival, behavior or physiology of common eider throughout the annual cycle [26], [27], [28], [35], [75]. Variation in individual decisions regarding movements, nest site selection, skipped breeding seasons and presence or absence of a floater population [38], [46], [76] may further contribute to inter-colony variation in demography. For common eider, weather probably affects: 1) adult survival but only during severe, impact years, most probably during harsher winters although it is doubtful that milder winters are entirely beneficial; 2) adult breeding propensity, winter via winter body condition or last year’s breeding conditions [31], [32], [33]. Furthermore, negative effects of rain or drought on duckling production would represent direct effects of weather on recruitment, although such effects are probably much localized [40].

Effects of weather may be dependent on adjacent topography. For instance, a dry summer may have quite different effects on the intertidal feeding habitat at low lying offshore islands, such as Flatey, in comparison with colonies close to mountain ranges, such as Þyrill or Hrísey, where there is abundant freshwater runoff from the mountains. Adjacent mountain ranges also may provide shelter from wind or influence amount of precipitation. During the duckling stage, shelter from wind is locally important in areas which face the open ocean (such as Fuglavík-Norðurkot, Flatey), whereas sheltered colonies inside fjords or inlets are often little affected. Lastly, breeding numbers are influenced by factors other than weather, including food availability, some of which may be weather-related. Furthermore, anthropogenic factors, such as protective efforts by the eider farmers, human depopulation events, changed farming practices, disturbance or farm abandonment can affect eider nest numbers.

## Supporting Information

Figure S1 (map) Numbers indicate locations of 17 colonies in Iceland which provided breeding numbers of common eider (*Somateria mollissima*) used in this study; numbers refer to numbers of colonies in Table 1. Names are the weather stations considered in this study.
(TIF)

Table S1 Principal components analysis of weather data from Stykkishólmur, West Iceland, for different periods where eider nests were counted in Iceland. The first two principal scores (PC) were used as indices of local weather. The highest loading for each variable in each analysis is indicated in bold.
(DOC)
