# Supplementary figures and images for: Relationships between Long-Term Demography and Weather in a Sub-Arctic Population of Common Eider
Source: PLoS One. 2013 Jun 21;8(6):e67093. doi: 10.1371/journal.pone.0067093 (PMC3689676; doi:10.1371/journal.pone.0067093)

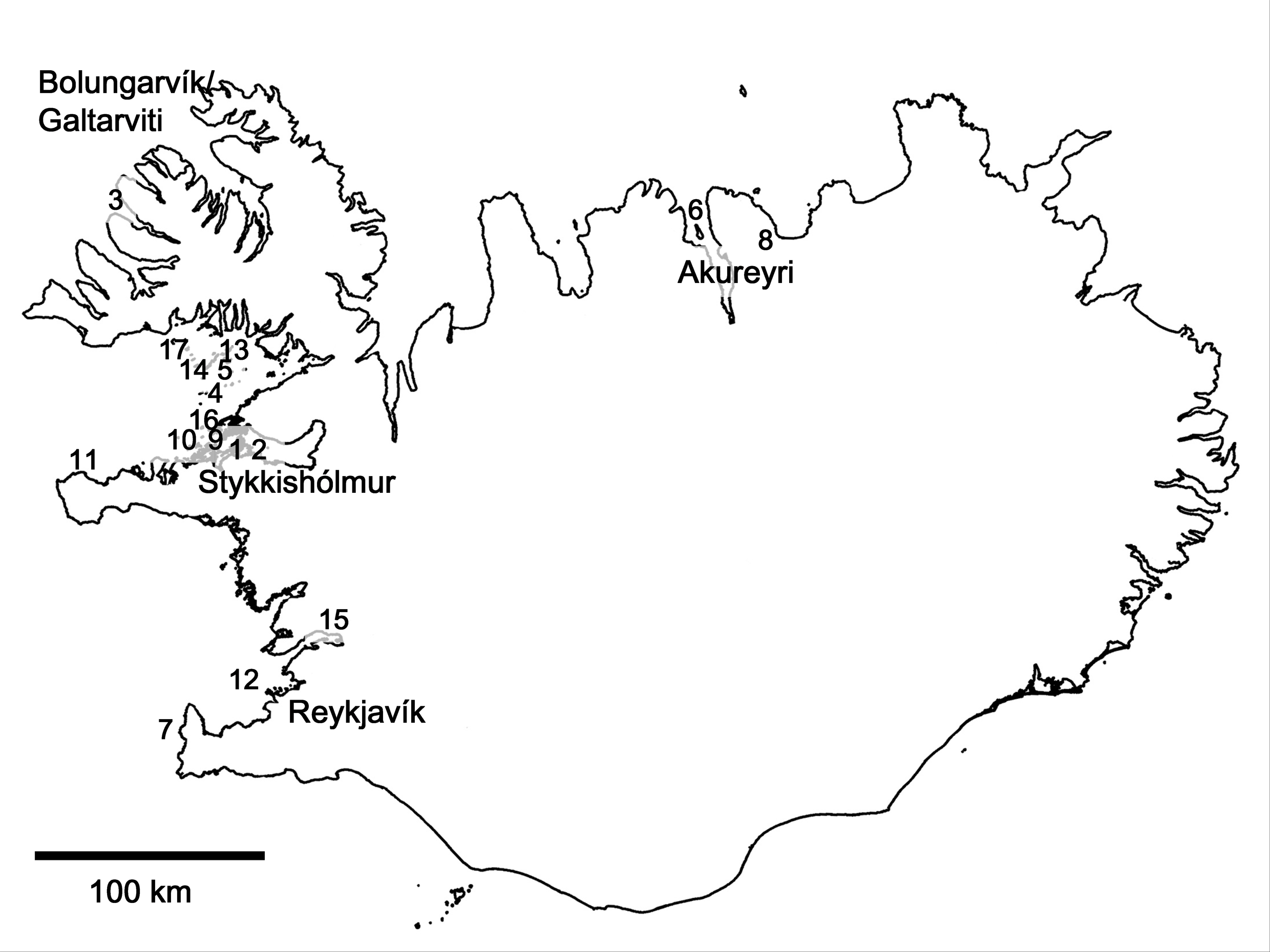

Supplement: Figure S1 — (map) Numbers indicate locations of 17 colonies in Iceland which provided breeding numbers of common eider (Somateria mollissima) used in this study; numbers refer to numbers of colonies in Table 1. Names are the weather stations considered in this study. (TIF) [file pone.0067093.s001.tif]
