# Supplementary material for: Relationships between Long-Term Demography and Weather in a Sub-Arctic Population of Common Eider
Source: PLoS One. 2013 Jun 21;8(6):e67093. doi: 10.1371/journal.pone.0067093 (PMC3689676; doi:10.1371/journal.pone.0067093)
Supplement: Table S1 — Principal components analysis of weather data from Stykkishólmur, West Iceland, for different periods where eider nests were counted in Iceland. The first two principal scores (PC) were used as indices of local weather. The highest loading for each variable in each analysis is indicated in bold. (DOC) [file pone.0067093.s002.doc]

Table S2. Principal components analysis of weather data from Stykkishólmur, West Iceland, for different periods where eider nests were counted in Iceland. The first two principal scores (PC) were used as indices of local weather. The highest loading for each variable in each analysis is indicated in bold.

1901-1930

| Weather variables | Summer-PC1 | Summer-PC2 | Fall-PC1 | Fall-PC2 | Winter-PC1 | Winter-PC2 | Spring-PC1 | Spring-PC2 |
| --- | --- | --- | --- | --- | --- | --- | --- | --- |
| Temperature (°C) | 0.12 | **0.98** | **0.72** | -0.10 | **0.65** | 0.42 | 0.20 | **0.92** |
| Precipitation (mm) | **0.71** | 0.04 | **0.68** | 0.32 | **0.72** | -0.08 | **-0.72** | -0.09 |
| Atmospheric pressure (Ppt) | **-0.70** | 0.20 | -0.16 | **0.94** | -0.24 | **0.90** | **0.67** | -0.37 |
| *Percentage (%) of variation explained within each season* | *52.4* | *33.6* | *42.5* | *34.0* | *47.2* | *34.8* | *45.6* | *34.5* |

1906-2007

| Weather variables | Summer-PC1 | Summer-PC2 | Fall-PC1 | Fall-PC2 | Winter-PC1 | Winter-PC2 | Spring-PC1 | Spring-PC2 |
| --- | --- | --- | --- | --- | --- | --- | --- | --- |
| Temperature (°C) | 0.44 | **0.78** | **0.67** | 0.33 | 0.54 | **0.64** | 0.35 | **0.87** |
| Precipitation (mm) | **0.71** | 0.00 | **0.71** | -0.06 | **0.73** | -0.03 | **0.71** | 0.00 |
| Atmospheric pressure (Ppt) | -0.55 | **0.62** | -0.19 | **0.94** | -0.42 | **-0.77** | **-0.61** | 0.50 |
| *Percentage (%) of variation explained within each season* | *52.2* | *33.6* | *45.3* | *33.9* | *50.4* | *36.2* | *45.0* | *33.2* |

1961-2007

| Weather variables | Summer-PC1 | Summer-PC2 | Fall-PC1 | Fall-PC2 | Winter-PC1 | Winter-PC2 | Spring-PC1 | Spring-PC2 |
| --- | --- | --- | --- | --- | --- | --- | --- | --- |
| Temperature (°C) | **0.69** | 0.24 | **0.74** | 0.27 | -0.17 | **0.73** | **0.55** | 0.25 |
| Precipitation (mm) | 0.00 | **0.76** | 0.16 | **0.81** | 0.46 | **0.56** | **0.60** | 0.15 |
| Atmospheric pressure (Ppt) | 0.29 | **-0.61** | 0.22 | -0.24 | **-0.66** | -0.12 | **-0.54** | 0.07 |
| Wind speed (m/s) | **-0.66** | -0.01 | **-0.61** | 0.46 | **0.57** | -0.37 | -0.21 | **0.95** |
| *Percentage (%) of variation explained within each season* | *31.9* | *29.3* | *32.0* | *29.8* | *43.0* | *36.0* | *37.5* | *24.7* |

1977-2007

| Weather variables | Summer-PC1 | Summer-PC2 | Fall-PC1 | Fall-PC2 | Winter-PC1 | Winter-PC2 | Spring-PC1 | Spring-PC2 |
| --- | --- | --- | --- | --- | --- | --- | --- | --- |
| Temperature (°C) | **0.65** | 0.05 | **0.73** | -0.14 | -0.23 | **0.76** | **0.54** | -0.11 |
| Precipitation (mm) | 0.41 | **-0.54** | **0.56** | 0.60 | 0.44 | **0.62** | **0.60** | 0.11 |
| Atmospheric pressure (Ppt) | 0.06 | **0.81** | -0.13 | -0.31 | **-0.64** | -0.02 | **-0.54** | -0.40 |
| Wind speed (m/s) | **-0.64** | -0.21 | -0.37 | **0.73** | **0.58** | -0.18 | -0.25 | **0.90** |
| *Percentage (%) of variation explained within each season* | *40.2* | *28.3* | *34.1* | *27.9* | *46.7* | *33.5* | *43.7* | *24.9* |
